# Ultrafast beam pattern modulation by superposition of chirped optical vortex pulses

**DOI:** 10.1038/s41598-022-18145-4

**Published:** 2022-09-02

**Authors:** Asami Honda, Keisaku Yamane, Kohei Iwasa, Kazuhiko Oka, Yasunori Toda, Ryuji Morita

**Affiliations:** 1 Department of Applied Physics, Hokkaido University, Kita-13, Nishi-8, Kita-ku, Sapporo, 060-8628 Japan; 2 Faculty of Science and Technology, Hirosaki University, 3 Bunkyo-cho, Hirosaki, 036-8561 Japan

**Keywords:** Optics and photonics, Physics

## Abstract

As an extension of pulse shaping techniques using the space–time coupling of ultrashort pulses or chirped pulses, we demonstrated the ultrafast beam pattern modulation by the superposition of chirped optical vortex pulses with orthogonal spatial modes. The stable and robust modulations with a modulation frequency of sub-THz were carried out by using the precise phase control technique of the constituent pulses in both the spatial and time/frequency domains. The performed modulations were ultrafast ring-shaped optical lattice modulation with 2, 4 and 6 petals, and beam pattern modulations in the radial direction. The simple linear fringe modulation was also demonstrated with chirped spatially Gaussian pulses. While the input pulse energy of the pulses to be modulated was 360 $\upmu$J, the output pulse energy of the modulated pulses was 115 $\upmu$J with the conversion efficiency of $\sim$ 32%. Demonstrating the superposition of orthogonal spatial modes in several ways, this ultrafast beam pattern modulation technique with high intensity can be applicable to the spatially coherent excitation of quasi-particles or collective excitation of charge and spin with dynamic degrees of freedom. Furthermore, we analyzed the Poynting vector and OAM of the composed chirped OV pulses. Although the ring-shaped optical lattice composed of OV pulse with topological charges of $\pm \, \ell$ is rotated in a sub-THz frequency, the net orbital angular momentum (OAM) averaged over one optical period is found to be negligible. Hence, it is necessary to require careful attention to the application of the OAM transfer interaction with matter by employing such rotating ring-shaped optical lattices.

## Introduction

Optical vortices (OVs), such as Laguerre–Gaussian modes, whose spatial phase depends on the azimuthal coordinate, have attracted much attention because of their unique properties. Possessing a phase singularity on their beam center, they carry the orbital angular momentum (OAM) proportional to the topological charge $\ell$, which is expressed by the value of the line integral of the spatial phase around the singular point. Their intensity profile in the beam cross-section is annular or in doughnut shape^1^. Owing to these properties, they are used in the various applications such as laser processing^2–13^, laser trapping/manipulation^14^, quantum information processing^15–19^, classical communications^20–23^, super-resolution microscopy^24–26^, and nonlinear spectroscopy^27–30^. In order to generate optical vortices, techniques by use of spiral phase plates^31^, space-variant wave plates^27^ or spatial light modulators (SLMs)^32^ have been employed. While the methods using phase or wave plates are comparably simple but lack the flexibility to change the topological charges of OVs, the scheme using SLMs tends to be slightly complicated but have an advantage of topological charge controllability.

In early works on OVs, cw or quasi-cw lasers have been used as light sources; recently, in addition to cw or quasi-cw lasers, pulsed or ultrashort pulsed lasers have been employed. However, OV generation and its application have been done by just modulation the spatial phase of laser pulses in almost all research. The technique of precise phase control in the frequency or temporal domain simultaneously with that in the spatial domain has not been fully carried out so far, except in our previous research^33,34^. By using the space-time coupling of ultrashort pulses or chirped pulses, pulse shaping^35^ or polarization pulse shaping^36,37^ in the 4-*f* system has been realized and applied to quantum control^38,39^. As an extension of such a technique, we demonstrate ultrafast beam pattern modulation by employing chirped OV pulses with space variant phase. While this technique of ultrafast beam pattern modulation is too fast to interact with real particles like micro beads, it has the potential to open the way to the applications such as sophisticated laser processing^4,8,9^, spatially controlled coherent excitation of quasi-particle (phonon, polariton, magnon)^40,41^ and spatially tailored plasma excitation^42^. It is useful for collective excitation of charge and spin with dynamic degrees of freedom^43^ where high-speed modulation is possible by using charge (spin) liquid crystals instead of common molecular liquid crystals. For example, it can be used for optical control of nematic electronic states (1D and 2D structures) appearing in iron-based superconductors^44^ and high-$T_\text {c}$ cuprate superconductors, and topological defects in chiral magnetic materials^45^.

In the present paper, we performed the ultrafast beam pattern modulation by the superposition of chirped OV pulses with orthogonal spatial modes. Stable and robust modulations with the frequency of a sub-THz were demonstrated by using the precise phase control technique of the pulses in both the spatial and frequency domain. The performed ultrafast modulations were ring-shaped optical lattice rotation and beam modulation in the radial direction or linear direction. Concerning the ring-shaped optical lattice rotations, they have been demonstrated with the ration speed of $\sim$ Hz, tens of MHz, and GHz, respectively, by mechanical rotation of plates^46^, acousto-optic modulator (AOM)^47^, and electro-optic modulator (EOM)^46^. In our previous study^34^, ultrafast ring-shaped optical lattice rotation with a frequency of sub-THz has been demonstrated. However, use of the polarizers, wave plates and high-order retarder hampered flexible beam pattern generation. In contrast to this, instead of the passive phase modulation plates, the present study employed a spatial phase modulator of the beams in addition to the comparatively low loss Sagnac interferometer-type system to combine beams. Additionally, the conversion efficiency of the ring-shaped optical lattice beam from the input beam was low as 50% at most in principle. The present method, therefore, enables stable and robust ultrafast beam modulation with pattern flexibility as well as high intensity. This method demonstrates the superposition of orthogonal spatial modes in several ways. In addition, we analyzed the Poynting vector and OAM of the composed chirped OV pulses. Although the ring-shaped optical lattice composed of OV pulse with topological charges $\pm\, \ell$ is rotated in a sub-THz frequency, the net orbital angular momentum (OAM) averaged over one optical period is found to be negligible. Nevertheless, such an ultrafast beam pattern modulation technique with high intensity can be applicable to the coherent excitation of quasi-particles such as phonon-polariton, exciton polariton and magnon, which has a fast response time. When the propagation velocity matching with the spatial modulation velocity is achieved, coherent enhancement of quasi-particle or collective excitation will be expected.

## Concept of ultrafast beam pattern modulation

First we consider the concept of ultrafast beam pattern modulation with the superposition of ultrashort pulses propagating in the *z* direction, as expressed by

1 $$\begin{aligned} {\textbf {E}}_1({\textbf {r}},t)&= \bar{{\textbf {E}}}_1({\textbf {r}},t) \exp \bigg [\mathrm{i}(kz -\omega _0 t +\psi _1)\bigg ], \end{aligned}$$

2 $$\begin{aligned} {\textbf {E}}_2({\textbf {r}},t)&= \bar{{\textbf {E}}}_2({\textbf {r}},t) \exp \bigg [\mathrm{i}(kz -\omega _0 t+\psi _2)\bigg ]. \end{aligned}$$

Here, $\bar{{\textbf {E}}}_1({\textbf {r}},t)$ and $\bar{{\textbf {E}}}_2({\textbf {r}},t)$ are slowly-varying envelopes of the electric fields, and ${\textbf {r}}$, $\omega _0$ and *t* respectively represents the spatial coordinates, center angular frequency and time. The phases of the pulse are represented by $\psi _1$ and $\psi _2$. The wave number is *k*.

The intensity pattern *I* of the interference between electric fields of the two pulses is proportional to

3 $$\begin{aligned} I \propto \bigg | {\textbf {E}}_1({\textbf {r}},t) +{\textbf {E}}_2({\textbf {r}},t) \bigg |^2&= \bigg | \bar{{\textbf {E}}}_1({\textbf {r}},t) \bigg |^2 + \bigg | \bar{{\textbf {E}}}_2({\textbf {r}},t) \bigg |^2 + 2 \text {Re} \bigg (\bar{{\textbf {E}}}_1({\textbf {r}},t) \cdot \bar{{\textbf {E}}}_2^*({\textbf {r}},t) \exp [\mathrm{i}( \psi _1-\psi _2) ]\bigg ), \end{aligned}$$

where $\text {Re}(\cdot )$ stands for the real part of the complex value. For linearly chirped optical pulses whose center angular frequency is $\omega _0$ and chirp coefficient *C*, its instantaneous angular frequency $\omega (t)$ is given by

4 $$\begin{aligned} \omega (t)= \omega _0+Ct. \end{aligned}$$

From the relationship ${\displaystyle \omega (t) = -\frac{\partial \psi }{\partial t}}$ between the phase $\psi$ of the electric field and the instantaneous frequency $\omega (t)$, the electric fields of the two pulses with a linear chirp coefficient are expressed by

5 $$\begin{aligned} {\textbf {E}}_1({\textbf {r}}, t)&= \bar{{\textbf {E}}}_1({\textbf {r}},t) \exp \bigg [\mathrm{i}(kz -\omega _0 t- C t^2/2)\bigg ], \end{aligned}$$

6 $$\begin{aligned} {\textbf {E}}_2({\textbf {r}}, t)&= \bar{{\textbf {E}}}_2({\textbf {r}},t) \exp \bigg [\mathrm{i}(kz -\omega _0 t- C t^2/2)\bigg ]. \end{aligned}$$

When the temporal interval $\tau _\text {CP}$ between the chirped pulses is introduced, the intensity pattern *I* of the interference Eq. (3) is rewritten by

7 $$\begin{aligned} I \propto \bigg | \bar{{\textbf {E}}}_1({\textbf {r}},t) \bigg |^2 + \bigg | \bar{{\textbf {E}}}_2({\textbf {r}},t-\tau _\text {CP}) \bigg |^2 + 2 \text {Re} \bigg ( \bar{{\textbf {E}}}_1({\textbf {r}},t) \cdot \bar{{\textbf {E}}}_2^*({\textbf {r}},t-\tau _\text {CP}) \exp \bigg [- \mathrm{i}\bigg (C\tau _\text {CP} t +\omega _0 \tau _\text {CP}-C\tau _\text {CP}^2/2\bigg ) \bigg ] \bigg ). \end{aligned}$$

The slowly-varying envelope of the electric field $\bar{{\textbf {E}}}_j({\textbf {r}},t)$ ($j=1$ or 2) of OV pulses with the radial index *p* and azimuthal index $\ell$ is expressed by

8 $$\begin{aligned} \bar{{\textbf {E}}}_j({\textbf {r}},t) = {\textbf {e}}_j\, {{\mathcal {E}}}_j(t) \bigg [ \frac{\sqrt{2} \rho }{ w(z)} \bigg ]^{|\ell _j|} L_p^{|\ell _j|}\bigg ( \frac{2\rho ^2}{w(z)^2} \bigg ) \frac{w_0}{w(z)} \exp \bigg [- \frac{\rho ^2}{w(z)^2} + \mathrm{i}\frac{k\rho ^2}{2R(z)} +\mathrm{i}\ell _j \phi -\mathrm{i}\Phi _\mathrm{G} (z) \bigg ], \end{aligned}$$

where $\rho$, $\phi$ and *z* denote the cylindrical coordinates, ${\textbf {e}}_j$ ($j=1$ or 2) is the polarization vector, ${{\mathcal {E}}}_j(t)$ ($j=1$ or 2) represents a temporal pulse shape function, and $L_p^{|\ell |}(x)$ is the generalized Laguerre polynomial defined by

9 $$\begin{aligned} L_p^{|\ell |}(x)=\sum _{r=0}^p (-1)^r \bigg ( \begin{array}{c} p+|\ell | \\ p-r \end{array} \bigg ) \frac{x^r}{r!}. \end{aligned}$$

Parameters *R*(*z*) and *w*(*z*) denote the radius of curvature of wavefronts and the beam size at a propagation distance *z*, as expressed by

10 $$\begin{aligned} R(z) = \bigg (z_\mathrm{R}^2+z^2\bigg )/z, \qquad w(z)= w_0 \sqrt{1+z^2/z_\mathrm{R}^2}, \end{aligned}$$

with the Rayleigh range

11 $$\begin{aligned} z_\mathrm{R}= k w_0^2/2. \end{aligned}$$

The constant $w_0$ is the beam waist. The parameter $\Phi _\mathrm{G}$ denotes Gouy phase, which is known to be an additional phase shift for a focused and propagated beam, differing from that for a plane wave. It is given by

12 $$\begin{aligned} \Phi _\mathrm{G}(z) = (2p+|\ell |+1 )\Phi (z) \equiv (2p+|\ell |+1 ){\arctan } (z/z_\mathrm{R}), \end{aligned}$$

where $\Phi (z)$ is the fundamental Gouy phase for the Gaussian beam.

Modes of Laguerre–Gaussian beam are expressed by the indices $p, \ell$ in the radial and azimuthal directions, respectively. In the case where a pair of pulse with a linear chirp coefficient the interference term in Eq. (7) between two OV pulses having a linear chirp coefficient with mode indices $(p, \ell )= (p_1, \ell _1)$ and $(p_2, \ell _2)$ is expressed by

13 $$ \begin{aligned}  & 2{\text{Re}}(\overline{{\mathbf{E}}} _{1} ({\mathbf{r}},t) \cdot \overline{{\mathbf{E}}} _{2}^{*} ({\mathbf{r}},t - \tau _{{{\text{CP}}}} )\exp [ - {\text{i}}(C\tau _{{{\text{CP}}}} t + \omega _{0} \tau _{{{\text{CP}}}} - C\tau _{{{\text{CP}}}}^{2} /2)]) \hfill \\&\quad  \propto 2\bigg [\frac{{\sqrt 2 \rho }}{{w(z)}}\bigg ]^{{|\ell _{1} | + |\ell _{2} |}} L_{{p_{1} }}^{{|\ell _{1} |}} \bigg (\frac{{2\rho ^{2} }}{{w(z)^{2} }}\bigg )L_{{p_{2} }}^{{|\ell _{2} |}} \bigg (\frac{{2\rho ^{2} }}{{w(z)^{2} }}\bigg ){\text{Re}}({\mathbf{e}}_{1} \cdot {\mathbf{e}}_{2}^{*} \, {\mathcal{E}}_{1} (t){\mathcal{E}}_{2}^{*} (t - \tau _{{{\text{CP}}}} )\exp \{ {\text{i}}[(\ell _{1} - \ell _{2} )\phi - C\tau _{{{\text{CP}}}} t - \omega _{0} \tau _{{{\text{CP}}}} + C\tau _{{{\text{CP}}}}^{2} /2]\} ). \hfill \\ \end{aligned} $$

**Figure 1:**
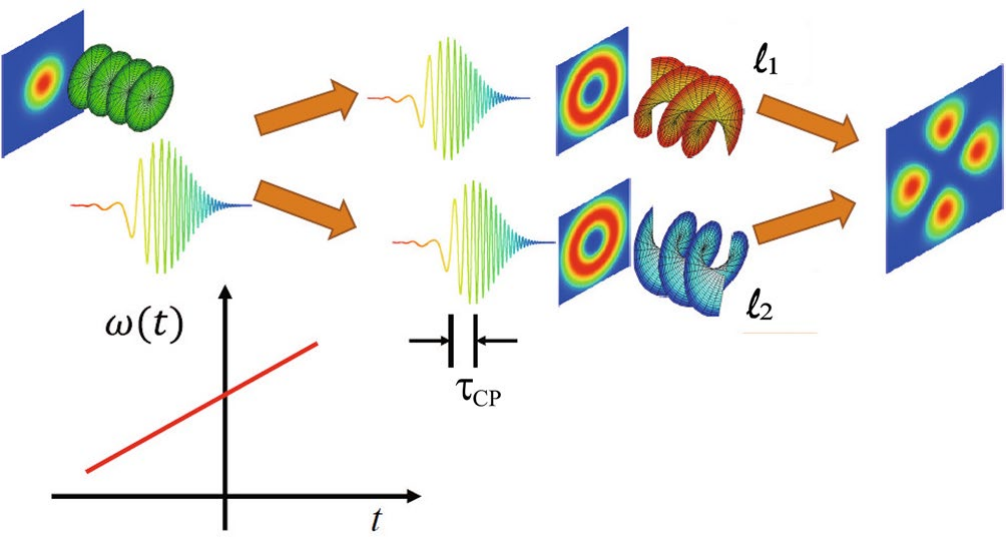
Schematic drawing of ultrafast spatial mode modulation by using chirped pulses of structured light (the case of the superposition of two OVs). $\tau_\text {CP}$: delay time between chirped pulses, $\omega (t)$: instantaneous frequency, $\ell _1, \ell _2$: topological charges of OV.

Schematic drawing of the superposition of a pair of chirped OV pulses with a time delay $\tau_\text {CP}$ is shown in Fig. 1.

## Results and discussion

### Ultrafast rotation of ring-shaped optical lattices in the azimuthal direction

Equation (13) indicates that the phase difference between the two OV pulses with azimuthal indices $\ell _1$ and $\ell _2$ is essentially given by $(\ell _1-\ell _2) \phi -C\tau _\text {CP} t -\omega _0 \tau _\text {CP}+C\tau _\text {CP}^2/2$ depending on the azimuthal coordinate $\phi$ and time *t*. Therefore, they create the $|\ell _1-\ell _2|$-fold ring-shaped optical lattice and the $|\ell _1-\ell _2|$-fold ring-shaped optical lattice rotates in the azimuth direction with a rotation speed of $|{C \tau _\text {CP}}/(\ell _1-\ell _2)|$. Ultrafast rotation of the ring-shaped optical lattice with time *t* is achieved by setting the typical chirp coefficient *C* of $\sim$ tens THz/ps and the delay $\tau _\text {CP}$ of $\sim$ sub ps.

**Figure 2:**
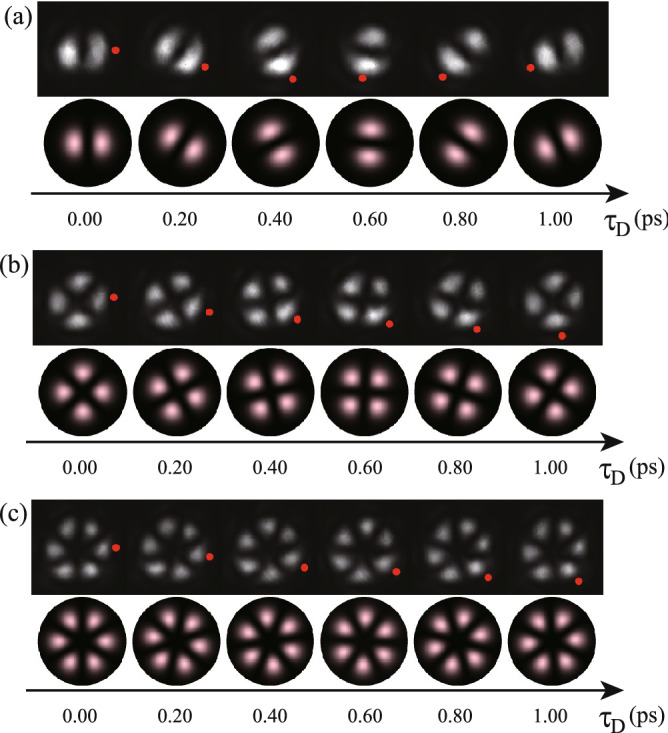
Ultrafast ring-shaped optical lattice rotation by the superposition of two chirped $p=0$ OV pulses with topological charge pairs of $\ell _1$ and $\ell _2$. The time-divided spatial patterns of SFG recorded on the CMOS camera at the nominal time delay $\tau _\text {D}$ between the generated pulse and the reference pulse of 0.00, 0.20, 0.40. 0.60, 0.80 and 1.00 ps; (**a**) $(\ell _1, \ell _2)=(1, -1)$, (**b**) $(\ell _1, \ell _2)=(2, -\,2)$, and (**c**) $(\ell _1, \ell _2)=(3, -\,3)$. Red points in a petal are guide for the eye. Experimental and simulated results are respectively at the top and bottom in (**a–c**).

First we superposed two chirped $p = 0$ OV pulses with topological charge of $(\ell _1, \ell _2)=(1, -1)$ and thereby obtained ring-shaped optical lattice rotation with twofold petal-like patterns. The sum frequency generation (SFG) was performed from pattern modulated beams and spatially Gaussian beam with a pulse duration of $\sim$ 100 fs as a reference. The time-divided spatial patterns of SFG were recorded on the CMOS camera at the time delay $\tau _\text {D}$ between the generated pulse and the reference pulse of 0.00, 0.20, 0.40. 0.60, 0.80 and 1.00 ps, as indicated at the top of Fig. 2a (see also Supplementary Movie S1). In similar manner, we performed the ring-shaped optical lattice rotation by superposing of two chirped $p=0$ OV pulses with topological charge pairs of $(\ell _1, \ell _2)=(2, -2)$ and $(\ell _1, \ell _2)=(3, -3)$. The time-divided SFG patterns recorded on the CMOS camera are respectively shown at the top of Fig. 2b,c (see also Supplementary Movies S2, S3).

**Figure 3:**
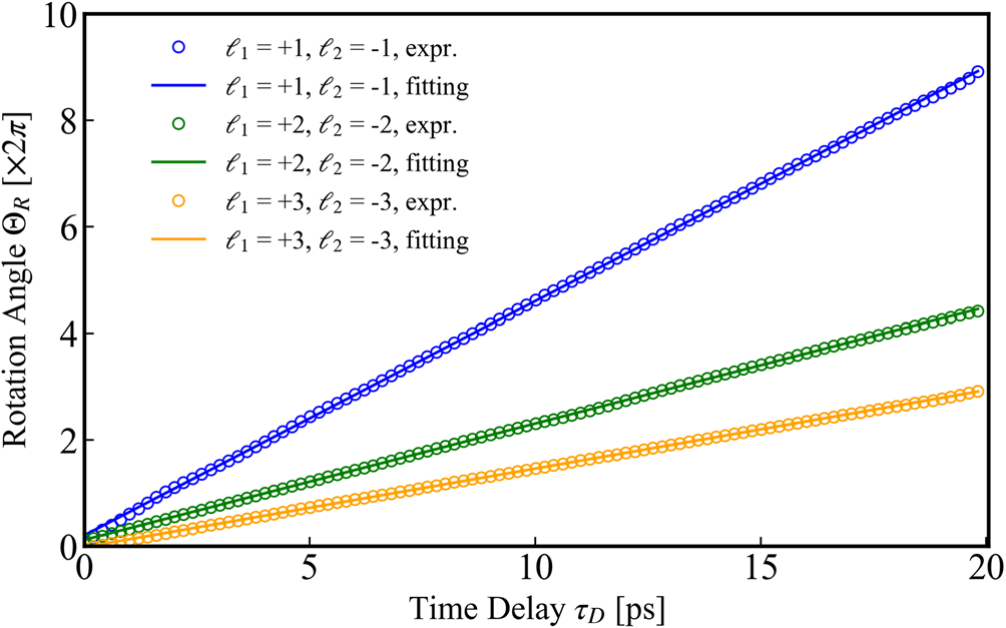
The rotation angle $\Theta _\text {R}$ of ring-shaped optical lattices as a function of time delay $\tau _\text {D}$.

The rotational angles $\Theta _\text {R}$ experimentally measured clockwise from the patterns at $\tau _\text {D}=0$ in Fig. 2a–c were plotted in Fig. 3 as a function of the delay $\tau _\text {D}$. The rotational angle $\Theta _\text {R}$ of the generated beams was evaluated from the temporal evolution of the beam intensity at a point in the beam cross section. The measured rotational angles are well fitted by the linear function of the delay $\tau _\text {D}$ from 0 to $\sim$ 20 ps, within an error of $\sim$ 3.6%. This indicates the stable ring-shaped optical lattice rotation or beam pattern modulation continues for $\sim 20$ ps in our optical system. The rotational angular frequencies $\Omega _\text {R}\equiv \Theta _\text {R}/\tau _\text {D}$ for the chirped pairs of $(\ell _1, \ell _2)= (1, -1), (2,-2)$ and $(3,-3)$ are respectively evaluated to be $0.87\pi$, $0.44\pi$, and $0.31\pi$ rad/ps or rad$\cdot$THz. The ratio of $\Omega _\text {R}$ for $(\ell _1, \ell _2)= (1, -1), (2,-2)$ and $(3,-3)$ pairs are evaluated to be 2.8:1.4:1.0. It is inversely proportional to the ratio of topological charge difference between chirped pulse pairs 2:4:6, being consistent the theory $\Omega _\text {R} =|{C \tau _\text {CP}}/(\ell _1-\ell _2)|$ as described in the subsection above.

The corresponding ultrafast ring-shaped optical lattice rotations were numerically simulated. The calculated results are shown at the bottom of Fig. 2 for the superposition of (a) $(\ell _1, \ell _2)= (1, -1)$ chirped vortex pulse pair with the rotational angular frequency $\Omega _\text {R}=0.87\pi$ rad/ps, (b) $(\ell _1, \ell _2)= (2, -2)$ chirped vortex pulse pair with the rotational angular frequency $\Omega _\text {R}=0.44\pi$ rad/ps, and (c) $(\ell _1, \ell _2)= (3, -3)$ chirped vortex pulse pair with the rotational angular frequency $\Omega _\text {R}=0.31\pi$ rad/ps, respectively. The experimentally obtained rotational patterns well agree with the calculated results, while somewhat deviation was due to spatial asymmetry of the used beams. The chirped coefficient is evaluated to be 7.7 ps$^{-2}$ from the fact that the interval $\tau _\text {CP}$ of the chirped pulse pair was set to be 0.73 ps in the system.

**Figure 4:**
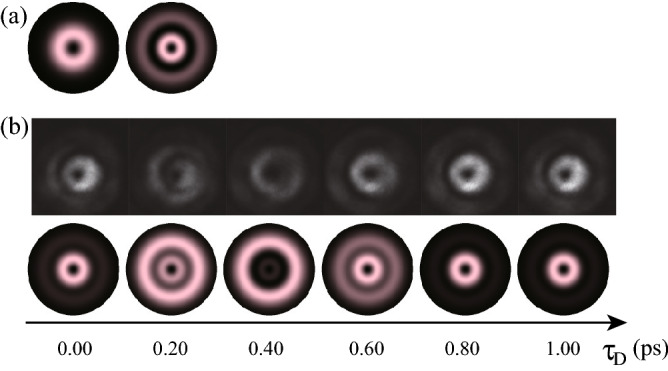
Ultrafast beam pattern modulation by the superposition of two chirped $\ell =1$ OV pulses with the radial indices of $(p_1, p_2) = (0,1)$. (**a**) Constituent OV beam patterns of $p_1=0$ and $p_2=1$ with $\ell =1$. (**b**) The time-divided spatial patterns of SFG recorded on the CMOS camera at the nominal time delay $\tau _\text {D}$ between the generated pulse and the reference pulse of 0.00, 0.20, 0.40. 0.60, 0.80 and 1.00 ps. Experimental and simulated results are respectively at the top and bottom in (**b**).

**Figure 5:**
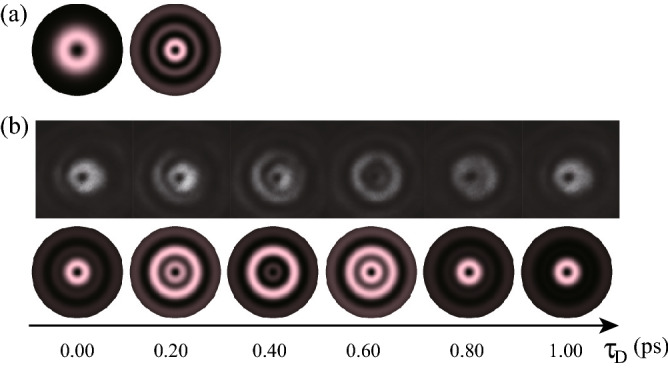
Ultrafast beam pattern modulation by the superposition of two chirped $\ell =1$ OV pulses with the radial indices of $(p_1,p_2) = (0,2)$. (**a**) Constituent OV beam patterns of $p_1=0$ and $p_2=2$ with $\ell =1$. (**b**) The time-divided spatial patterns of SFG recorded on the CMOS camera at the nominal time delay $\tau _\text {D}$ between the generated pulse and the reference pulse of 0.00, 0.20, 0.40. 0.60, 0.80 and 1.00 ps. Experimental and simulated results are respectively at the top and bottom in (**b**).

### Ultrafast beam pattern modulation in the radial direction

In the case where $\ell _1=\ell _2(=\ell )$ in Eq. (13), the phase difference between the two OV pulses is governed by $-C\tau _\text {CP}t-\omega _0 \tau _\text {CP} +C\tau _\text {CP}^2/2$. When the two OV pulses with the same azimuthal index $\ell$ but different radial indices $p_1$ and $p_2$, the spatial modes of them are different in the radial direction. Thus, in this case, different spatial modes with phase difference of $-C\tau _\text {CP} t$ depending on time *t*, gives the beam pattern modulation in the radial direction.

Second, we carried out ultrafast beam pattern modulation by the superposition of chirped $\ell =1$ OV pulses with the radial indices of $(p_1,p_2) = (0,1)$ and $(p_1,p_2) = (0,2)$, as respectively shown in Fig. 4 (see also Supplementary Movie S4) and Fig. 5 (see also Supplementary Movie S5). For $(p_1,p_2)=(0,1)$ with $\ell =1$, the constituent beam patterns are shown in Fig. 4a. The $p=0$ mode has a single ring located at a radius of $\rho \simeq \, 0.707 w$ where *w* is the beam radius at the camera ; the $p=1$ mode has dual rings at $\rho \simeq \, 0.468 w$, and 1.51*w* with relative phase difference of $\pi$. By the superposition of chirped OV pulses with $p=0$ and $p=1$ modes, the gradual mode-switching or beam pattern modulation effectively in radial direction is obtained using $\pi$ phase difference and relative phase modulation of chirped pulses. Figure 4b, top and bottom, respectively shows the corresponding experimental and simulated results.

For $(p_1,p_2) = (0,2)$ with $\ell =1$, the constituent beam patterns are shown in Fig. 5a. The $p=0$ mode has a single ring located at $\rho \simeq 0.707 w$ where *w* is the beam radius at the camera ; the $p=2$ mode has triple rings at $\rho \simeq \, 0.379 w, 1.13 w$ and 2.02*w* with adjacent relative phase difference of $\pi$. Similarly, by the superposition of chirped OV pulses with $p=0$ and $p=2$ modes, beam pattern modulation effectively in radial direction is obtained using $\pi$ phase difference and relative phase modulation of chirped pulses. Figure 5b, top and bottom, respectively shows the corresponding experimental and simulated results. In both cases of $(p_1,p_2) = (0,1)$ and (0, 2), the experimental and calculated results, being consistent with each other except the deviation due to the spatial asymmetry of the used beams, indicate ultrafast beam pattern modulation effectively in the radial direction with a modulation frequency of sub-THz.

### Linear beam pattern modulation

In this subsubsection, we consider a simpler case of the superposition of not chirped OV pulses but chirped pulses with a spatially plane profile. When such two pulses propagating in ${\textbf {k}}_1$ and ${\textbf {k}}_2$ directions, as expressed by

14 $$\begin{aligned} {\textbf {E}}_1({\textbf {r}}, t)&= \bar{{\textbf {E}}}_1({\textbf {r}},t) \exp \bigg [\mathrm{i}\bigg ({\textbf {k}}_1\cdot {\textbf {r}} -\omega _0 t- C t^2/2\bigg )\bigg ], \end{aligned}$$

15 $$\begin{aligned} {\textbf {E}}_2({\textbf {r}}, t)&= \bar{{\textbf {E}}}_2({\textbf {r}},t) \exp \bigg [\mathrm{i}\bigg ({\textbf {k}}_2\cdot {\textbf {r}} -\omega _0 t- C t^2/2\bigg )\bigg ], \end{aligned}$$

are interfered with a crossing angle, the interference term is given by

16 $$ \begin{gathered}  2{\text{Re}}(\overline{{\mathbf{E}}} _{1} ({\mathbf{r}},t) \cdot \overline{{\mathbf{E}}} _{2}^{*} ({\mathbf{r}},t - \tau _{{{\text{CP}}}} )\exp [ - {\text{i}}(C\tau _{{{\text{CP}}}} t + \omega _{0} \tau _{{{\text{CP}}}} - C\tau _{{{\text{CP}}}}^{2} /2)]) \hfill \\  \propto {\text{Re}}({\mathbf{e}}_{1} \cdot {\mathbf{e}}_{2}^{*} \, A_{1} (t)A_{2}^{*} (t - \tau _{{{\text{CP}}}} )\exp \{ {\text{i}}[({\mathbf{k}}_{1} - {\mathbf{k}}_{2} ) \cdot {\mathbf{r}} - C\tau _{{{\text{CP}}}} t - \omega _{0} \tau _{{{\text{CP}}}} + C\tau _{{{\text{CP}}}}^{2} /2]\} ), \hfill \\ \end{gathered} $$

the parameters are defined similarly as in the above, except ${\textbf {k}}_1$ and ${\textbf {k}}_2$, which are propagation vector in different directions. In this case, plane-like spatial profiles with phase difference of $({\textbf {k}}_1-{\textbf {k}}_2) \cdot {\textbf {r}} -C\tau _\text {CP} t$ depending on ${\textbf {r}}$ and *t*, yield the movement or pattern modulation of linear fringes in the ${\textbf {k}}_1-{\textbf {k}}_2$ direction. The speed of moving is expressed by $|C\tau _\text {CP}|/|{\textbf {k}}_1-{\textbf {k}}_2|\simeq |C\tau _\text {CP}| \lambda _0/ (2\pi \alpha )$, where $\lambda _0$ is the center wavelength ($\lambda _0 \equiv 2\pi c/\omega _0$, *c*: velocity of light in vacuum) and $\alpha$ is the beam crossing angle.

**Figure 6:**
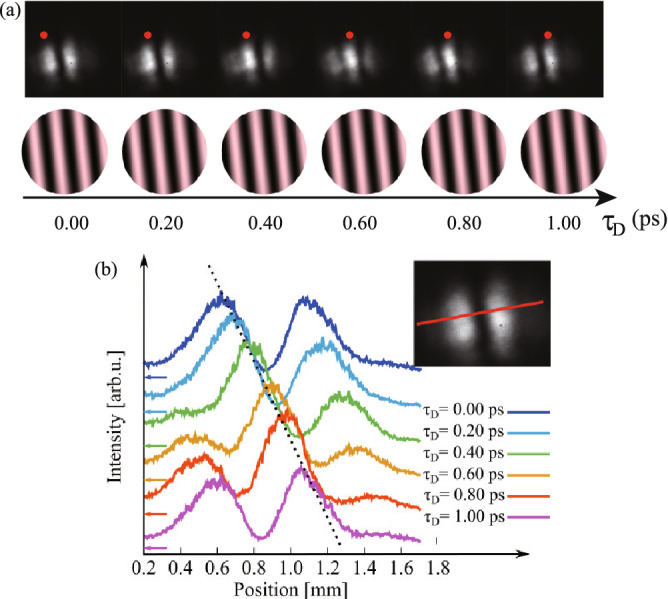
Ultrafast beam pattern modulation by the superposition of two chirped pulses with spatially Gaussian modes having a small crossing angle. (**a**) The time-divided spatial patterns of SFG recorded on the CMOS camera at the nominal time delay $\tau _\text {D}$ between the generated pulse and the reference pulse of 0.00, 0.20, 0.40. 0.60, 0.80 and 1.00 ps. Red points in a bright line are guides for the eye. Experimental and simulated results are respectively at the top and bottom. (**b**) Intensity profiles of the modulated patterns along the red line (perpendicular to the fringes) as shown at the top right, with a parameter of the time delay $\tau _\text {D}$. Dotted line is a guide for the eye.

Third, not for the superposition of OV pulses but of spatially uniform phase optical pulses, the ultrafast beam pattern modulation was demonstrated. In this case, two chirped pulses with spatially Gaussian modes were superimposed with a small crossing angle. The ultrafast modulated beam patterns in the horizontal direction are shown at the top of Fig. 6a, together with the corresponding numerical results at the bottom of Fig. 6a (see also Supplementary Movie S6). Figure 6b indicates the intensity profiles of the modulated patterns along the red line (perpendicular to the fringes) as shown at the top right of the figure, with a parameter of the time delay $\tau _\text {D}$ between the generated pulse and the reference pulse. Experimental results clearly show linear fringe shifting, agreeing well with the simulated results. Vibration frequencies from optical components or air fluctuation are far from the modulation frequency. Even when the vibrations are imposed on the time-divided SFG patterns, they do not affect ultrafast applications of modulated beams.

### Poynting vector and angular momenta of optical vortex with the superposition of ultrashort chirped pulses

Here, we consider angular momentum for the beam composed of chirped ultrashort OV pulses. A circularly polarized vector potential ${\textbf {A}}$ is given by

17 $$\begin{aligned} {\textbf {A}}({\textbf {r}},t)&= \frac{1}{2\sqrt{2}} ({\textbf {e}}_x +\mathrm{i}\sigma {\textbf {e}}_y) [G(t) {{\mathcal {U}}}_1({\textbf {r}}) + G(t-\tau _\text {CP}) {{\mathcal {U}}}_2({\textbf {r}}) ] + \text {c.c.} \nonumber \\&=\frac{1}{2\sqrt{2}} \exp (\mathrm{i}\sigma \phi ) ({\textbf {e}}_\rho +\mathrm{i}\sigma {\textbf {e}}_\phi ) [G(t) {{\mathcal {U}}}_1({\textbf {r}}) + G(t-\tau _\text {CP}) {{\mathcal {U}}}_2({\textbf {r}}) ] + \text {c.c.}, \end{aligned}$$

18 $$\begin{aligned} G(t)&= \exp (-\mathrm{i}\omega _0 t-\mathrm{i}C t^2/2) g(t), \end{aligned}$$

19 $$\begin{aligned} {{\mathcal {U}}}_1({\textbf {r}})&= \exp (\mathrm{i}kz) U_1({\textbf {r}}), \end{aligned}$$

20 $$\begin{aligned} {{\mathcal {U}}}_2({\textbf {r}})&= \exp (\mathrm{i}kz) U_2({\textbf {r}}), \end{aligned}$$

where ${\textbf {e}}_x$ and ${\textbf {e}}_y$ are respectively the unit vector in the *x* and *y*-directions, and ${\textbf {r}}, t, \omega _0, C$, and $\tau _\text {CP}$ are the position vector, time, center frequency, chirp coefficient, and time delay, respectively. The cylindrical coordinates are expressed by $\rho , \phi$ and *z* and ${\textbf {e}}_\rho , {\textbf {e}}_\phi$ and ${\textbf {e}}_z$ are the unit vectors in $\rho , \phi$ and *z*-directions, respectively. The temporal envelope function of the pulse is given by a real function *g*(*t*), for example, a Gaussian or hyperbolic-secant shape; the function *G*(*t*) is defined to be *g*(*t*) multiplied by a frequency chirped exponential factor of $\exp (-\mathrm{i}\omega _0 t-\mathrm{i}C t^2/2)$. The parameter $\sigma$ represents the sense of circular polarization; $\sigma =+\,1$ and $-\,1$ respectively indicate left and right circular polarizations.

The magnetic field ${\textbf {B}}$ is given by ${\textbf {B}}({\textbf {r}},t)=\text {rot}\, {\textbf {A}}({\textbf {r}},t)$, and the electric field is calculated from the relationship of ${\partial {\textbf {E}}({\textbf {r}},t)}/{\partial t}= c^2 \text {rot}\, {\textbf {B}}({\textbf {r}},t)$. Thereby, the instantaneous Poynting vector ${\textbf {S}}({\textbf {r}},t)$, time-averaged over one optical period $T=2\pi /\omega _0$, for the paraxial beam with the slowly-varying envelope approximation in time domain is calculated to be^48^

21 $$\begin{aligned} {\textbf {S}}({\textbf {r}},t)&=\frac{1}{T} \epsilon _0 c^2 \int _{t-T/2}^{t+T/2} {}[{\textbf {E}}({\textbf {r}},t')\times {\textbf {B}}({\textbf {r}},t')] \, {\mathrm {d}} t' {}={\textbf {e}}_\rho S_\rho ({{\textbf {r}},t}) +{\textbf {e}}_\phi S_\phi ({{\textbf {r}},t}) +{\textbf {e}}_z S_z({{\textbf {r}},t}), \end{aligned}$$

22 $$\begin{aligned} S_\rho ({{\textbf {r}},t})&= -\mathrm{i}\frac{\omega _0}{8} \bigg \{|G(t)|^2 \bigg [ U_1^*({\textbf {r}}) \partial _\rho U_1({\textbf {r}})-U_1({\textbf {r}}) \partial _\rho U_1^*({\textbf {r}})\bigg ] +\bigg |G(t-\tau _\text {CP})\bigg |^2 \bigg [ U_2^*({\textbf {r}}) \partial _\rho U_2({\textbf {r}})-U_2({\textbf {r}}) \partial _\rho U_2^*({\textbf {r}})\bigg ] \nonumber \\&\quad + G(t) G^*(t-\tau _\text {CP}) [ U_2^*({\textbf {r}}) \partial _\rho U_1({\textbf {r}})-U_1({\textbf {r}}) \partial _\rho U_2^*({\textbf {r}})] + G^*(t) G(t-\tau _\text {CP}) [ U_1^*({\textbf {r}}) \partial _\rho U_2({\textbf {r}})-U_2({\textbf {r}}) \partial _\rho U_1^*({\textbf {r}})] \bigg \} \nonumber \\&\quad + \text {c.c.}, \end{aligned}$$

23 $$\begin{aligned} S_\phi ({{\textbf {r}},t})&= -\frac{\sigma \omega _0}{8} \bigg \{|G(t)|^2 \partial _\rho |U_1({\textbf {r}})|^2 +|G(t-\tau _\text {CP})|^2 \partial _\rho |U_2({\textbf {r}})|^2 \nonumber \\&\quad + G(t) G^*(t-\tau _\text {CP}) \partial _\rho [ U_1({\textbf {r}}) U_2^*({\textbf {r}})] + G^*(t) G(t-\tau _\text {CP}) \partial _\rho [U_1^*({\textbf {r}}) U_2({\textbf {r}})] \bigg \} \nonumber \\&\quad -\,\mathrm{i}\frac{\omega _0}{8\rho } \bigg \{|G(t)|^2 [ U_1^*({\textbf {r}}) \partial _\phi U_1({\textbf {r}})-U_1({\textbf {r}}) \partial _\phi U_1^*({\textbf {r}})] +|G(t-\tau )|^2 [U_2^*({\textbf {r}}) \partial _\phi U_2({\textbf {r}})-U_2({\textbf {r}}) \partial _\phi U_2^*({\textbf {r}})] \nonumber \\&\quad + G(t) G^*(t-\tau ) [ U_2^*({\textbf {r}}) \partial _\phi U_1({\textbf {r}})-U_1({\textbf {r}}) \partial _\phi U_2^*({\textbf {r}})] + G^*(t) G(t-\tau ) [U_1^*({\textbf {r}}) \partial _\phi U_2({\textbf {r}})-U_2({\textbf {r}}) \partial _\phi U_1^*({\textbf {r}})] \} \nonumber\\&\quad + \text {c.c.}, \end{aligned}$$

24 $$\begin{aligned} S_z({{\textbf {r}},t}) = \frac{\omega _0^2}{2c}&\bigg | G(t) U_1({{\textbf {r}}}) +G(t-\tau _\text {CP}) U_2({\textbf {r}}) \bigg |^2, \end{aligned}$$

where $\epsilon _0$ and *c* are the permittivity and the speed of light in the vacuum and the differential operator ${\partial }/{\partial X}$ is expressed by $\partial _X$. Here, we assume that $|\omega _0| \gg |C t_\text {p}|$, where $t_\text {p}$ is the pulse duration (for example, the full width at half maximum of $g(t)^2$). As previously reported^1^, the first component $S_\rho$ represents the flux density in $\rho$-direction. For the second component $S_\phi$, the terms with a factor of $-\sigma \omega _0/8$ are the spin angular momentum (SAM) terms; the terms with a factor of $-\mathrm{i}\omega _0/8 \rho$ indicate the orbital angular momentum (OAM) terms corresponding to the flux density in $\phi$-direction. The third component $S_z$ is proportional to the linear momentum in *z*-direction. However, it should be noted that, in this case of the composite beam, the components of ${\textbf {S}}$ are governed by the spatial and temporal overlaps of the two constituent pulsed beams.

Since $\overline{{\textbf {p}}}({{\textbf {r}},t}) \equiv {\textbf {S}}({\textbf {r}},t)/c^2$ represents the instantaneous linear momentum density, the instantaneous OAM density $\overline{{\textbf {L}}}({{\textbf {r}},t})$ is given by

25 $$\begin{aligned} \overline{{\textbf {L}}}({{\textbf {r}},t}) =&{\textbf {r}} \times \overline{{\textbf {p}}}({{\textbf {r}},t}) ={\textbf {r}} \times {\textbf {S}}({\textbf {r}},t)/c^2=\epsilon _0 {\textbf {r}} \times [{\textbf {E}}({\textbf {r}},t)\times {\textbf {B}}({\textbf {r}},t)] \nonumber \\ =&\{ {\textbf {e}}_\rho [-z S_\phi ({{\textbf {r}},t})] +{\textbf {e}}_\phi [zS_\rho ({{\textbf {r}},t})- \rho S_z({{\textbf {r}},t})] +{\textbf {e}}_z \rho S_\phi ({{\textbf {r}},t}) \} /c^2. \end{aligned}$$

The overlapping term of temporal functions is

26 $$\begin{aligned} G(t)G^*(t-\tau _\text {CP}) =\exp \bigg [-\mathrm{i}(C\tau _\text {CP} t + \omega _0\tau _\text {CP} -C\tau _\text {CP}^2/2)\bigg ] g(t) g(t-\tau _\text {CP}). \end{aligned}$$

In the case for an OV pulse pair where

27 $$\begin{aligned} U_1({\textbf {r}})&= \exp (\mathrm{i}\ell _1 \phi ) u_1({\textbf {r}}), \end{aligned}$$

28 $$\begin{aligned} U_2({\textbf {r}})&= \exp (\mathrm{i}\ell _2 \phi ) u_2({\textbf {r}}), \end{aligned}$$

with a complex spatial function $u_j({\textbf {r}})$ $(j=1,2)$, the components of the instantaneous Poynting vector ${\textbf {S}}({\textbf {r}},t)$ are expressed by

29 $$\begin{aligned} S_\rho ({{\textbf {r}},t})&= -\mathrm{i}\frac{\omega _0}{8} \bigg \{g(t)^2 \bigg [ u_1^*({ \textbf {r}}) \partial _\rho u_1({\textbf {r}})-u_1({\textbf {r}}) \partial _\rho u_1^*({\textbf {r}})\bigg ] +g(t-\tau _\text {CP})^2 \bigg [ u_2^*({\textbf {r}}) \partial _\rho u_2({\textbf {r}})-u_2({\textbf {r}}) \partial _\rho u_2^*({\textbf {r}})\bigg ]\nonumber \\&\quad + \exp \bigg \{\mathrm{i}[(\ell _1-\ell _2)\phi -C\tau _\text {CP} t - \omega _0\tau _\text {CP} +C\tau _\text {CP}^2/2]\bigg \} g(t) g(t-\tau _\text {CP}) \bigg [ u_2^*({\textbf {r}}) \partial _\rho u_1({\textbf {r}})-u_1({\textbf {r}}) \partial _\rho u_2^*({\textbf {r}})\bigg ] \nonumber \\&\quad + \exp \bigg \{-\mathrm{i}[(\ell _1-\ell _2)\phi -C\tau _\text {CP} t - \omega _0\tau _\text {CP} +C\tau _\text {CP}^2/2]\bigg \} g(t) g(t-\tau _\text {CP}) \bigg [ u_1^*({\textbf {r}}) \partial _\rho u_2({\textbf {r}})-u_2({\textbf {r}}) \partial _\rho u_1^*({\textbf {r}})\bigg ] \bigg \} \nonumber \\&\quad +\text {c.c.}, \end{aligned}$$

30 $$\begin{aligned} S_\phi ({{\textbf {r}},t})&= -\frac{\sigma \omega _0}{4} \bigg \{g(t)^2 \partial _\rho |u_1({\textbf {r}})|^2 +g(t-\tau _\text {CP})^2 \partial _\rho |u_2({\textbf {r}})|^2 \nonumber \\&\quad + \exp \bigg \{\mathrm{i}[(\ell _1-\ell _2)\phi -C\tau _\text {CP} t - \omega _0\tau _\text {CP} +C\tau _\text {CP}^2/2]\bigg \} g(t) g(t-\tau _\text {CP}) \bigg [ u_1({\textbf {r}}) \partial _\rho u_2^*({\textbf {r}})+u_2^*({\textbf {r}}) \partial _\rho u_1({\textbf {r}})\bigg ] \nonumber \\&\quad + \exp \bigg \{-\mathrm{i}[(\ell _1-\ell _2)\phi -C\tau _\text {CP} t - \omega _0\tau _\text {CP} +C\tau _\text {CP}^2/2] \bigg \} g(t) g(t-\tau _\text {CP}) \bigg [ u_2({\textbf {r}}) \partial _\rho u_1^*({\textbf {r}})+u_1^*({\textbf {r}}) \partial _\rho u_2({\textbf {r}}) \bigg ] \bigg \} \nonumber \\&\quad +\frac{\omega _0}{4\rho } \bigg \{2 \ell _1 g(t)^2 |u_1({\textbf {r}})|^2 +2\ell _2 g(t-\tau _\text {CP})^2 |u_2({\textbf {r}})|^2 \nonumber \\&\quad + \exp \bigg \{\mathrm{i} [(\ell _1-\ell _2)\phi -C\tau _\text {CP} t - \omega _0\tau _\text {CP} +C\tau _\text {CP}^2/2 ]\bigg \} (\ell _1+\ell _2) g(t) g(t-\tau _\text {CP}) u_1({\textbf {r}}) u_2^*({\textbf {r}}) \nonumber \\&\quad + \exp \bigg \{-\mathrm{i}[(\ell _1-\ell _2)\phi -C\tau _\text {CP} t - \omega _0\tau _\text {CP} +C\tau _\text {CP}^2/2]\bigg \} (\ell _1+\ell _2) g(t) g(t-\tau _\text {CP}) u_1^*({\textbf {r}}) u_2({\textbf {r}}) \bigg \},\end{aligned}$$

31 $$\begin{aligned} S_z({{\textbf {r}},t})&= \frac{\omega _0^2}{2c}\bigg \{ g(t)^2 |u_1({\textbf {r}})|^2 +g(t-\tau _\text {CP})^2 |u_2({\textbf {r}})|^2 \nonumber \\&\quad + \exp \bigg \{\mathrm{i}[(\ell _1-\ell _2)\phi -C\tau _\text {CP} t - \omega _0\tau _\text {CP} +C\tau _\text {CP}^2/2]\bigg \} g(t) g(t-\tau _\text {CP}) u_1({\textbf {r}}) u_2^*({\textbf {r}}) \nonumber \\&\quad + \exp \bigg \{-\mathrm{i}[(\ell _1-\ell _2)\phi -C\tau _\text {CP} t - \omega _0\tau _\text {CP} +C\tau _\text {CP}^2/2]\bigg \} g(t) g(t-\tau _\text {CP}) u_1^*({\textbf {r}}) u_2({\textbf {r}}) \bigg \}. \end{aligned}$$

As mentioned in the above, the OAM of the composite beam $L_z\propto \rho S_\phi$ in the *z*-direction is governed by the spatial and temporal overlaps of the constituent pulsed beams. For the case of the synthesis of $\ell _1$ and $\ell _2$ vortex beams, the OAM is given by the terms of the individual pulses beams and the cross-terms (interference terms) of the constituent beams. It should be emphasized that, in particular, the interference terms vanish when $\ell _1+\ell _2=0$ from Eq. (30). Even for the rotational ring-shaped optical lattice owing to the interference with $\ell _1$ and $\ell _2 (= - \ell _1)$ vortex beams, the net OAM averaged over one optical period is negligible. Hence, it is necessary to require careful attention to the application of the OAM transfer interaction with matter by employing rotating ring-shaped optical lattices with $\ell _1+\ell _2\not = 0$.

## Concluding remarks

We generated chirped ultrafast OV pulses with precise phase control techniques in both the spatial and time/frequency domains. By employing the superposition of these chirped OV pulses with orthogonal spatial modes, we conducted the ultrafast beam pattern modulation. The stable and robust modulation with a modulation frequency of sub-THz were demonstrated. The performed modulations were ultrafast ring-shaped optical lattice modulation with 2, 4 and 6 petals and beam pattern modulations in the radial direction. The simple linear fringe modulation was also demonstrated with chirped spatially Gaussian pulses. We employed a spatial phase modulator of the beams in addition to the comparatively low loss Sagnac interferometer-type system to combine beams. For the input pulse energy of the pulses to be modulated was 360 $\upmu$J, the output pulse energy of the modulated pulses was 115 $\upmu$J. Therefore, the conversion efficiency of the beam pattern modulation was evaluated to be $\sim$ 32%, which was about 10 times higher than that in our previous work^34^.

Moreover, we analyzed the Poynting vector and OAM of the superposed OV pulses. It is shown that, even when the ring-shaped optical lattices with $\ell _1+\ell _2\not =0$ are rotated with a sub-THz frequency, they have no net OAM averaged over one optical period. Therefore, it is indicated that we should pay careful attention to the application of the OAM transfer interaction with matter by employing such rotating ring-shaped optical lattices.

The superposition of orthogonal spatial modes with our ultrafast beam pattern modulation technique can be extended for other spatial modes, and it will be a useful tool for the space dependent excitation, or spectroscopy of quasi-particles or collective excitation in materials. When we control the spatial modulation velocity to match the propagation velocity, coherent enhancement of quasi-particle excitation will be expected.

## Methods

### Experimental setup

**Figure 7:**
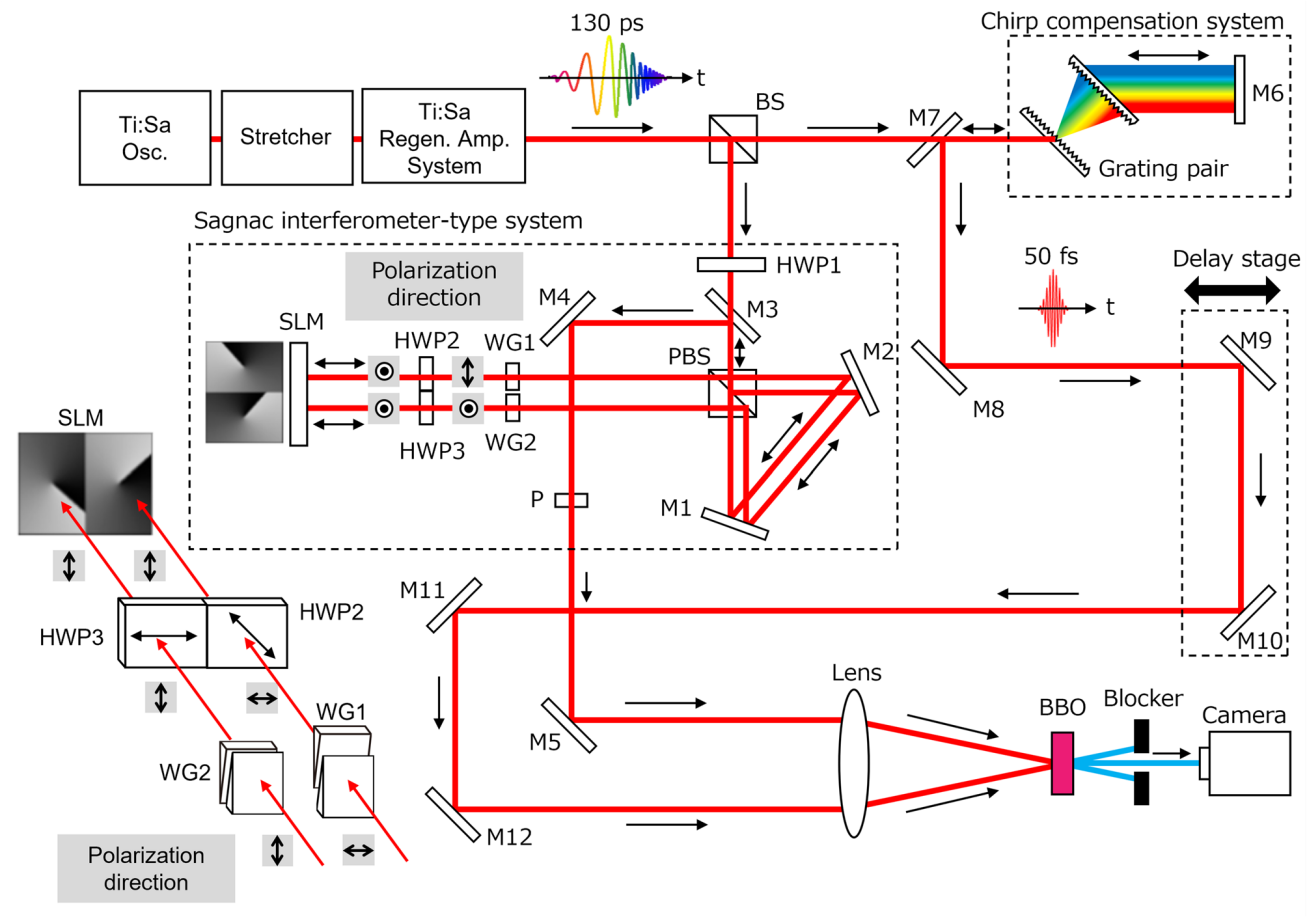
Experimental setup for ultrafast beam pattern modulation. BS, beam splitter; M1-12, mirrors; PBS, polarizing beam splitter; SLM, spatial light modulator; WG1–2, wedged glass; HWP1–3, half-wave plates; P, polarizer; BBO, $\beta$-BaB$_2$O$_4$ crystal. M3 is a vertically-shifted mirror for directing the phase-modulated beams by SLM into the time-divided spatial pattern monitoring part. M7 is a vertically-shifted mirror for guiding the reference beam after chirp compensation by a pair of grating into the time-divided spatial pattern monitoring part. A pair of wedged glasses are used in each optical path in the Sagnac interferometer-type system to give a time delay of the chirped pulse pair, avoiding lateral beam shifts. Half-wave plates with fast axes in horizontal and 45$^\circ$ directions, making vertically-polarized light on SLM, are employed to minimize efficiency difference of the diffraction in two paths. Schematic of wedged glasses, half-wave plates and SLM with polarization relations are depicted in the lower-left.

The experimental setup is shown in Fig. 7. The light source was a home-made Ti:sapphire regenerative amplifier system (center wavelength $\lambda \simeq$ 800 nm and bandwidth (full width at half-maximum) $\Delta \lambda \simeq$ 100 nm, repetition rate of 1 kHz) without pulse compression. The pulse from the regenerative amplifier was horizontally polarized and strongly chirped without chirp compensation so that its pulse duration was $\sim$ 130 ps. The chirped pulse is divided into two by a beam splitter (BS). One is guided to a Sagnac interferometer-type beam combining system after passing through a half-wave plate to rotate a polarization direction by 45$^\circ$. The other is directed to a chirp compensation system to generate a reference pulse with a duration of $\sim$ 50 fs for a spatio-temporal measurement system of the pattern modulated beams. The chirp compensation system was composed of a transmission grating pair (grating density 1200 lines/mm) and a mirror.

In the Sagnac interferometer-type beam combining system, the input pulse was sent to a polarizing beam splitter (PBS) and divided into two orthogonally polarized, that is, horizontally and vertically polarized pulses. A pair of wedged glasses (WG1 and 2) are used in each optical path in the Sagnac interferometer-type system to give a time delay $\tau _\text {CP}$ of the chirped pulse pair, avoiding lateral beam shifts. Half-wave plates HWP2 and 3 with fast axes in horizontal and 45$^\circ$ directions, making vertically-polarized light on SLM, are employed to minimize efficiency difference of the diffraction in two paths. Schematic of wedged glasses(WG1,2), half-wave plates (HWP2, 3) and SLM with polarization relations are depicted in the lower-left in Fig. 7.

The individual pulses were thereafter modulated independently with computer-generated hologram (CGH) patterns by using a reflective-type spatial light modulator. The two pulses were going back and re-combined at the PBS. The recombined pulse from the Sagnac interferometer-type system and the reference pulse, both directed into the spatio-temporal measurement system, were focused by convex lenses (focal lengths $f=$ 150 mm and $\sim$ 1000 mm, respectively) into $\beta$-barium borate (BBO) crystal (type I, thickness 100 $\upmu$m) for the sum-frequency generation (SFG). By monitoring the spatial intensity patterns of the SFG with a CMOS (2048$\times$1536 pixels, pixel size 3.45 $\times$ 3.45 $\upmu$m$^2$) camera as a function of the temporal delay $\tau _\text {D}$ between the recombined pulse and the reference pulse, the time-resolved spatial intensity profile of the spatially modulated pulse (the recombined pulse) was obtained.

While the input pulse energy of the pulses to be modulated was 360 $\upmu$J, the output pulse energy of the modulated pulses was 115 $\upmu$J. Therefore, the conversion efficiency of the beam pattern modulation was evaluated to be $\sim$ 32%, which was about 10 times higher than that in our previous work^34^.

## Supplementary Information

Supplementary Legends.

Supplementary Movie 1.

Supplementary Movie 2.

Supplementary Movie 3.

Supplementary Movie 4.

Supplementary Movie 5.

Supplementary Movie 6.

## Data Availability

The datasets used and/or analyzed during the current study available from the corresponding author on reasonable request.
