# Publisher Correction: Ultrafast beam pattern modulation by superposition of chirped optical vortex pulses

**DOI:** 10.1038/s41598-022-21946-2

**Published:** 2022-11-04

**Authors:** Asami Honda, Keisaku Yamane, Kohei Iwasa, Kazuhiko Oka, Yasunori Toda, Ryuji Morita

**Affiliations:** 1 Department of Applied Physics, Hokkaido University, Kita‑13, Nishi‑8, Kita‑ku, Sapporo, 060‑8628 Japan; 2 Faculty of Science and Technology, Hirosaki University, 3 Bunkyo‑cho, Hirosaki, 036‑8561 Japan

Correction to: *Scientific Reports* 10.1038/s41598-022-18145-4, published online 02 September 2022

The original version of this Article contained errors in Equations 22, 29, and 30 where extra brackets were incorrectly added to Equations 22 and 29, and the brackets in Equation 30 were captured in the wrong font size. Additionally, value "**r**" in Equations 22, 29 and 30 was wrongly presented as "*r*".

Equation 22

$$\begin{aligned} S_\rho ({{ {r}},t})&= -\mathrm{i}\frac{\omega _0}{8} \bigg \{|G(t)|^2 \bigg [ U_1^*({ {r}}) \partial _\rho U_1({ {r}})-U_1({ {r}}) \partial _\rho U_1^*({ {r}})\bigg ] +\bigg |G(t-\tau _\text {CP})\bigg |^2 \bigg [ U_2^*({ {r}}) \partial _\rho U_2({ {r}})]-U_2({ {r}}) \partial _\rho U_2^*({ {r}})\bigg ] \nonumber \\&\quad + G(t) G^*(t-\tau _\text {CP}) [ U_2^*({ {r}}) \partial _\rho U_1({ {r}})-U_1({ {r}}) \partial _\rho U_2^*({ {r}})] + G^*(t) G(t-\tau _\text {CP}) [ U_1^*({ {r}}) \partial _\rho U_2({ {r}})-U_2({ {r}}) \partial _\rho U_1^*({ {r}})] \bigg \} \nonumber \\&\quad + \text {c.c.}, \end{aligned}$$

now reads:

$$\begin{aligned} S_\rho ({{\textbf {r}},t})&= -\mathrm{i}\frac{\omega _0}{8} \bigg \{|G(t)|^2 \bigg [ U_1^*({\textbf {r}}) \partial _\rho U_1({\textbf {r}})-U_1({\textbf {r}}) \partial _\rho U_1^*({\textbf {r}})\bigg ] +\bigg |G(t-\tau _\text {CP})\bigg |^2 \bigg [ U_2^*({\textbf {r}}) \partial _\rho U_2({\textbf {r}})-U_2({\textbf {r}}) \partial _\rho U_2^*({\textbf {r}})\bigg ] \nonumber \\&\quad + G(t) G^*(t-\tau _\text {CP}) [ U_2^*({\textbf {r}}) \partial _\rho U_1({\textbf {r}})-U_1({\textbf {r}}) \partial _\rho U_2^*({\textbf {r}})] + G^*(t) G(t-\tau _\text {CP}) [ U_1^*({\textbf {r}}) \partial _\rho U_2({\textbf {r}})-U_2({\textbf {r}}) \partial _\rho U_1^*({\textbf {r}})] \bigg \} \nonumber \\&\quad + \text {c.c.}, \end{aligned}$$

Equation 29

$$\begin{aligned} S_\rho ({{{r}},t})&= -\mathrm{i}\frac{\omega _0}{8} \bigg \{g(t)^2 \bigg [ u_1^*({ {r}}) \partial _\rho u_1({ {r}})-u_1({ {r}}) \partial _\rho u_1^*({ {r}})\bigg ] +g(t-\tau _\text {CP})^2 \bigg [ u_2^*({ {r}}) \partial _\rho u_2({ {r}})]-u_2({ {r}}) \partial _\rho u_2^*({ {r}})\bigg ]\nonumber \\&\quad + \exp \bigg \{\mathrm{i}[(\ell _1-\ell _2)\phi -C\tau _\text {CP} t - \omega _0\tau _\text {CP} +C\tau _\text {CP}^2/2]\bigg \} g(t) g(t-\tau _\text {CP}) \bigg [ u_2^*({ {r}}) \partial _\rho u_1({ {r}})-u_1({ {r}}) \partial _\rho u_2^*({ {r}})\bigg ] \nonumber \\&\quad + \exp \bigg \{-\mathrm{i}[(\ell _1-\ell _2)\phi -C\tau _\text {CP} t - \omega _0\tau _\text {CP} +C\tau _\text {CP}^2/2]\bigg \} g(t) g(t-\tau _\text {CP}) \bigg [ u_1^*({ {r}}) \partial _\rho u_2({ {r}})-u_2({ {r}}) \partial _\rho u_1^*({ {r}})\bigg ] \bigg \} \nonumber \\&\quad +\text {c.c.}, \end{aligned}$$

now reads:

$$\begin{aligned} S_\rho ({{\textbf {r}},t})&= -\mathrm{i}\frac{\omega _0}{8} \bigg \{g(t)^2 \bigg [ u_1^*({\textbf {r}}) \partial _\rho u_1({\textbf {r}})-u_1({\textbf {r}}) \partial _\rho u_1^*({\textbf {r}})\bigg ] +g(t-\tau _\text {CP})^2 \bigg [ u_2^*({\textbf {r}}) \partial _\rho u_2({\textbf {r}})-u_2({\textbf {r}}) \partial _\rho u_2^*({\textbf {r}})\bigg ]\nonumber \\&\quad + \exp \bigg \{\mathrm{i}[(\ell _1-\ell _2)\phi -C\tau _\text {CP} t - \omega _0\tau _\text {CP} +C\tau _\text {CP}^2/2]\bigg \} g(t) g(t-\tau _\text {CP}) \bigg [ u_2^*({\textbf {r}}) \partial _\rho u_1({\textbf {r}})-u_1({\textbf {r}}) \partial _\rho u_2^*({\textbf {r}})\bigg ] \nonumber \\&\quad + \exp \bigg \{-\mathrm{i}[(\ell _1-\ell _2)\phi -C\tau _\text {CP} t - \omega _0\tau _\text {CP} +C\tau _\text {CP}^2/2]\bigg \} g(t) g(t-\tau _\text {CP}) \bigg [ u_1^*({\textbf {r}}) \partial _\rho u_2({\textbf {r}})-u_2({\textbf {r}}) \partial _\rho u_1^*({\textbf {r}})\bigg ] \bigg \} \nonumber \\&\quad +\text {c.c.}, \end{aligned}$$

Equation 30

$$\begin{aligned} S_\phi ({{ {r}},t})&= -\frac{\sigma \omega _0}{4} \bigg \{g(t) \partial _\rho |u_1({ {r}})|^2 +g(t-\tau _\text {CP})^2 \partial _\rho |u_2({ {r}})|^2 \nonumber \\&\quad + \exp \bigg \{\mathrm{i}[(\ell _1-\ell _2)\phi -C\tau _\text {CP} t - \omega _0\tau _\text {CP} +C\tau _\text {CP}^2/2]\bigg \} g(t) g(t-\tau _\text {CP}) \bigg [ u_1({ {r}}) \partial _\rho u_2^*({ {r}})+u_2^*({ {r}}) \partial _\rho u_1({ {r}})\bigg ] \nonumber \\&\quad + \exp \bigg \{-\mathrm{i}[(\ell _1-\ell _2)\phi -C\tau _\text {CP} t - \omega _0\tau _\text {CP} +C\tau _\text {CP}^2/2]\} g(t) g(t-\tau _\text {CP}) [ u_2({ {r}}) \partial _\rho u_1^*({ {r}})+u_1^*({ {r}}) \partial _\rho u_2({ {r}})] \bigg \} \nonumber \\&\quad +\frac{\omega _0}{4\rho } \bigg \{2 \ell _1 g(t)^2 |u_1({ {r}})|^2 +2\ell _2 g(t-\tau _\text {CP})^2 |u_2({ {r}})|^2 \nonumber \\&\quad + \exp \bigg \{\mathrm{i}\bigg [(\ell _1-\ell _2)\phi -C\tau _\text {CP} t - \omega _0\tau _\text {CP} +C\tau _\text {CP}^2/2\bigg ]\bigg \} (\ell _1+\ell _2) g(t) g(t-\tau _\text {CP}) u_1({ {r}}) u_2^*({ {r}}) \nonumber \\&\quad + \exp \bigg \{-\mathrm{i}[(\ell _1-\ell _2)\phi -C\tau _\text {CP} t - \omega _0\tau _\text {CP} +C\tau _\text {CP}^2/2]\bigg \} (\ell _1+\ell _2) g(t) g(t-\tau _\text {CP}) u_1^*({ {r}}) u_2({ {r}}) \bigg \},\end{aligned}$$

now reads:

$$\begin{aligned} S_\phi ({{\textbf {r}},t})&= -\frac{\sigma \omega _0}{4} \bigg \{g(t) \partial _\rho |u_1({\textbf {r}})|^2 +g(t-\tau _\text {CP})^2 \partial _\rho |u_2({\textbf {r}})|^2 \nonumber \\&\quad + \exp \bigg \{\mathrm{i}[(\ell _1-\ell _2)\phi -C\tau _\text {CP} t - \omega _0\tau _\text {CP} +C\tau _\text {CP}^2/2]\bigg \} g(t) g(t-\tau _\text {CP}) \bigg [ u_1({\textbf {r}}) \partial _\rho u_2^*({\textbf {r}})+u_2^*({\textbf {r}}) \partial _\rho u_1({\textbf {r}})\bigg ] \nonumber \\&\quad + \exp \bigg \{-\mathrm{i}[(\ell _1-\ell _2)\phi -C\tau _\text {CP} t - \omega _0\tau _\text {CP} +C\tau _\text {CP}^2/2]\bigg\} g(t) g(t-\tau _\text {CP}) \bigg[ u_2({\textbf {r}}) \partial _\rho u_1^*({\textbf {r}})+u_1^*({\textbf {r}}) \partial _\rho u_2({\textbf {r}})\bigg] \bigg \} \nonumber \\&\quad +\frac{\omega _0}{4\rho } \bigg \{2 \ell _1 g(t)^2 |u_1({\textbf {r}})|^2 +2\ell _2 g(t-\tau _\text {CP})^2 |u_2({\textbf {r}})|^2 \nonumber \\&\quad + \exp \bigg \{\mathrm{i}[(\ell _1-\ell _2)\phi -C\tau _\text {CP} t - \omega _0\tau _\text {CP} +C\tau _\text {CP}^2/2]\bigg \} (\ell _1+\ell _2) g(t) g(t-\tau _\text {CP}) u_1({\textbf {r}}) u_2^*({\textbf {r}}) \nonumber \\&\quad + \exp \bigg \{-\mathrm{i}[(\ell _1-\ell _2)\phi -C\tau _\text {CP} t - \omega _0\tau _\text {CP} +C\tau _\text {CP}^2/2]\bigg \} (\ell _1+\ell _2) g(t) g(t-\tau _\text {CP}) u_1^*({\textbf {r}}) u_2({\textbf {r}}) \bigg \},\end{aligned}$$

The original Article has been corrected.

