# Supplementary material for: Ultrafast beam pattern modulation by superposition of chirped optical vortex pulses
Source: Sci Rep. 2022 Sep 2;12:14991. doi: 10.1038/s41598-022-18145-4 (PMC9440229; doi:10.1038/s41598-022-18145-4)
Supplement: Supplementary file 1 — Supplementary Legends. [file 41598_2022_18145_MOESM1_ESM.pdf]

LEGEND OF SUPPLEMENTARY MOVIES

Movie S1:
Movie of the experimental result of ultrafast ring-shaped optical lattice rotation by the superposition of two chirped $p = 0$ OV pulses with topological charge pairs of $\ell_1 = 1$ and $\ell_2 = -1$ (corresponding to Fig. 2(a)). The delay indicates the temporal delay $\tau_{\rm D}$ between the modulated beam pulse and the reference pulse.

Movie S2:
Movie of the experimental result of ultrafast ring-shaped optical lattice rotation by the superposition of two chirped $p = 0$ OV pulses with topological charge pairs of $\ell_1 = 2$ and $\ell_2 = -2$ (corresponding to Fig. 2(b)). The delay indicates the temporal delay $\tau_{\rm D}$ between the modulated beam pulse and the reference pulse.

Movie S3:
Movie of the experimental results of ultrafast ring-shaped optical lattice rotation by the superposition of two chirped $p = 0$ OV pulses with topological charge pairs of $\ell_1 = 3$ and $\ell_2 = -3$ (corresponding to Fig. 2(c)). The delay indicates the temporal delay $\tau_{\rm D}$ between the modulated beam pulse and the reference pulse.

Movie S4:
Movie of the experimental results of ultrafast beam pattern modulation by the superposition of two chirped $\ell = 1$ OV pulses with the radial indices of $(p_1, p_2) = (0, 1)$ (corresponding to Fig. 4(b)). The delay indicates the temporal delay $\tau_{\rm D}$ between the modulated beam pulse and the reference pulse.

Movie S5:
Movie of the experimental results of Ultrafast beam pattern modulation by the superposition of two chirped $\ell = 1$ OV pulses with the radial indices of $(p_1, p_2) = (0, 2)$ (corresponding to Fig. 5(b)). The delay indicates the temporal delay $\tau_{\rm D}$ between the modulated beam pulse and the reference pulse.

Movie S6:
Movie of the experimental results of ultrafast beam pattern modulation by the superposition of two chirped pulses with spatially Gaussian modes having a small crossing angle (corresponding to Fig. 6). The delay indicates the temporal delay $\tau_{\rm D}$ between the modulated beam pulse and the reference pulse.
