# Supplementary figures and images for: Ultrafast beam pattern modulation by superposition of chirped optical vortex pulses
Source: Sci Rep. 2022 Sep 2;12:14991. doi: 10.1038/s41598-022-18145-4 (PMC9440229; doi:10.1038/s41598-022-18145-4)

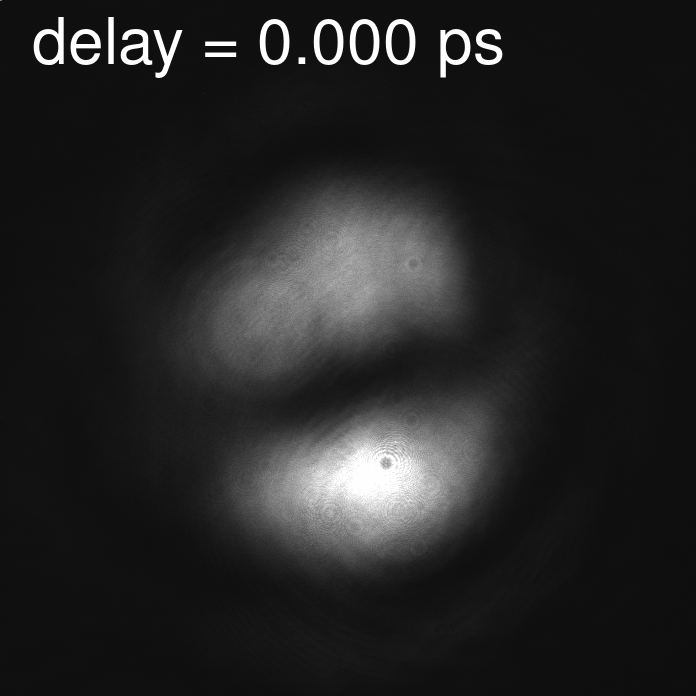

Supplement: Supplementary file 2 — Supplementary Movie 1. [file 41598_2022_18145_MOESM2_ESM.gif]

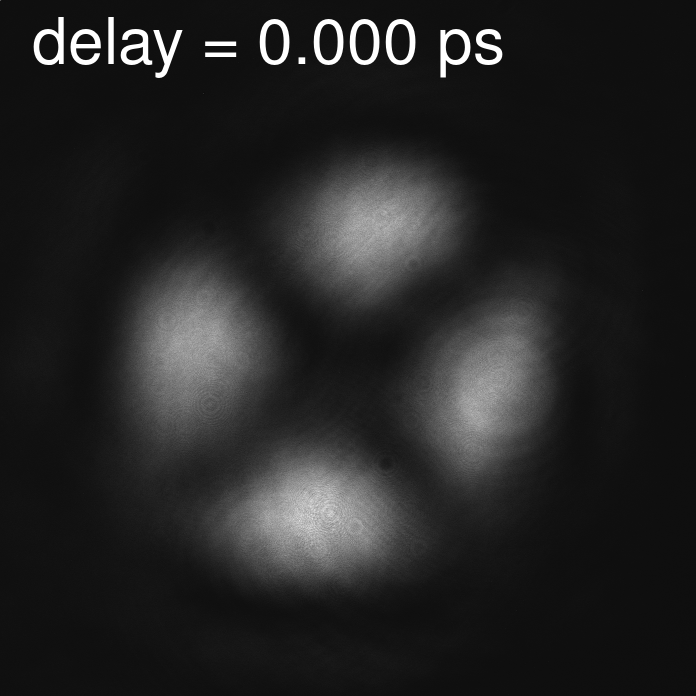

Supplement: Supplementary file 3 — Supplementary Movie 2. [file 41598_2022_18145_MOESM3_ESM.gif]

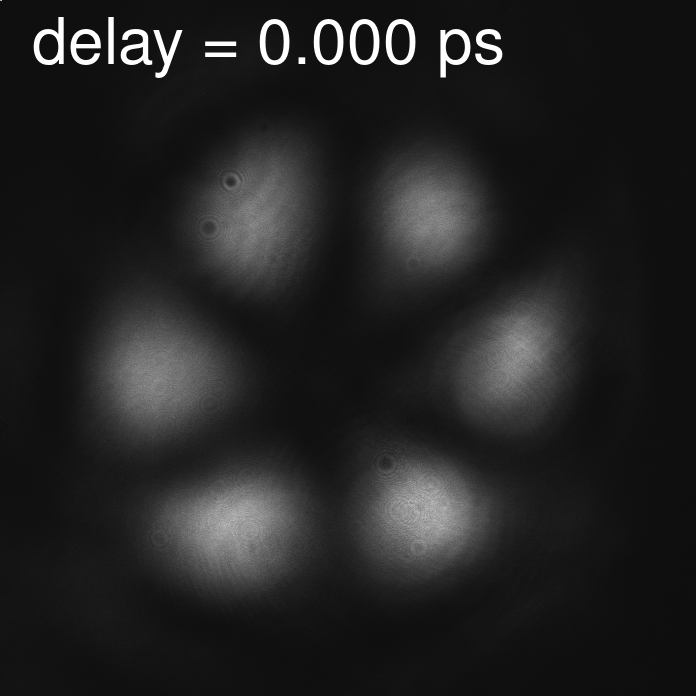

Supplement: Supplementary file 4 — Supplementary Movie 3. [file 41598_2022_18145_MOESM4_ESM.gif]

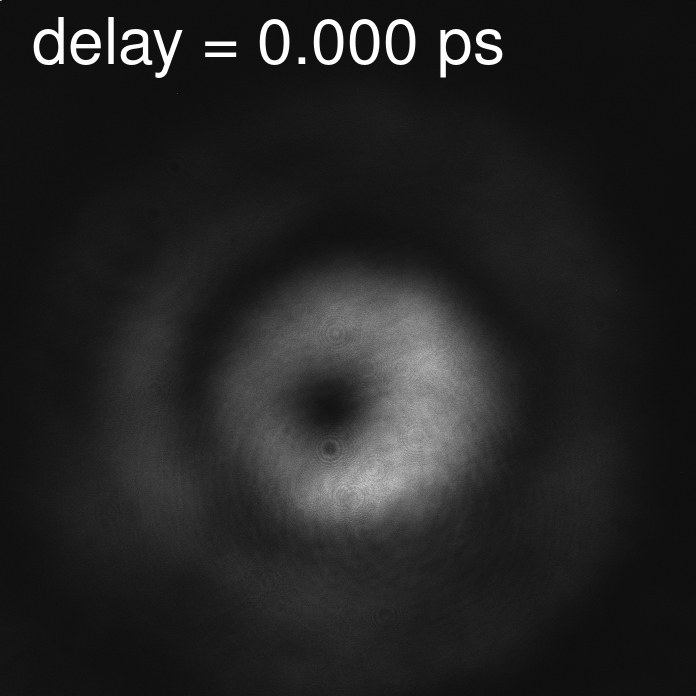

Supplement: Supplementary file 5 — Supplementary Movie 4. [file 41598_2022_18145_MOESM5_ESM.gif]

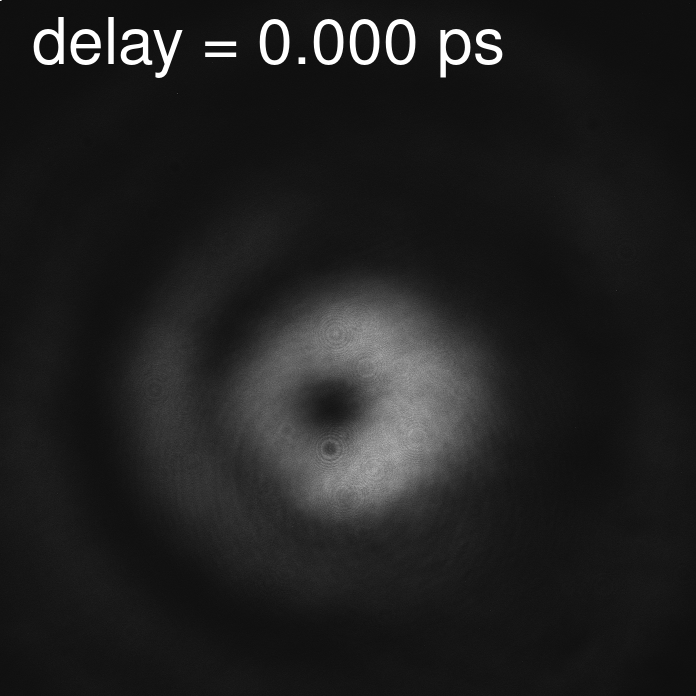

Supplement: Supplementary file 6 — Supplementary Movie 5. [file 41598_2022_18145_MOESM6_ESM.gif]

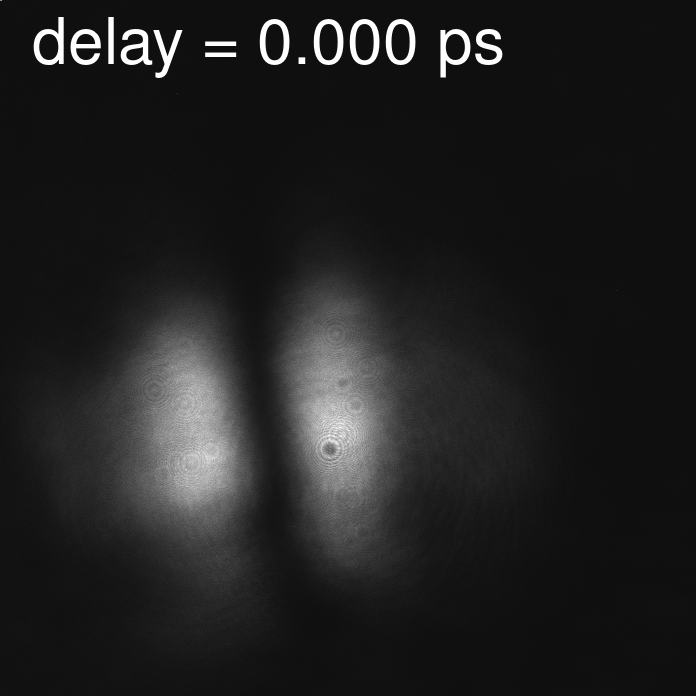

Supplement: Supplementary file 7 — Supplementary Movie 6. [file 41598_2022_18145_MOESM7_ESM.gif]
